# Editorial: Targeting major human fungal pathogens: novel insights into virulence and antifungal therapies

**DOI:** 10.3389/fcimb.2026.1832463

**Published:** 2026-03-31

**Authors:** Tianshu Sun, Hailong Li, Yuanwei Zhang

**Affiliations:** 1 Biobank, Institute of Clinical Medine, Peking Union Medical College Hospital(PUMCH), Chinese Academy of Medical Sciences and Peking Union Medical College, Beijing, China; 2 The First Affiliated Hospital of China Medical University, Shenyang, China; 3 College of Life Sciences, Nanjing Normal University, Nanjing, China

**Keywords:** anti-fungal drug developments, anti-fungal therapeutics, aspergillosis, candidiasis, cryptococcosis, fungal pathogenicity

The global burden of invasive fungal infections continues to escalate, driven by an expanding population of immunocompromised patients, climate-mediated emergence of new pathogens, and the relentless acquisition of multidrug resistance (Casadevall, 2022; Lockhart et al., 2023; Hoenigl et al., 2024). The World Health Organization’s 2022 fungal priority pathogens list underscored the urgency of this crisis by designating *Candida auris*, *Cryptococcus neoformans*, and azole-resistant *Aspergillus fumigatus* as critical threats (Casalini et al., 2024). This Research Topic was convened to capture the most promising molecular, translational, and technological advances that are reshaping how we understand and combat these organisms. The Research Topic unites four original research articles and eight authoritative reviews that collectively chart a path from basic discovery to next-generation therapeutics.

## Original discoveries: decoding pathogen armor—from natural product disruptors to metabolic and genomic vulnerabilities

Odabas et al. interrogate the lichen metabolome and identify lichesterinic acid, a paraconic acid, as a potent disruptor of *Candida albicans* and *Nakaseomyces glabratus* biofilms. Beyond *in vitro* efficacy, the compound demonstrates mammalian compatibility and curative activity in a Galleria infection model, positioning natural products as a still-underexploited reservoir of anti-biofilm agents. For primary mould pathogens, Yasin et al. dissect the *Aspergillus flavus* sugar transportome. Deletion of two major facilitator superfamily transporters (G4B84_001982 and G4B84_005374) simultaneously cripples aflatoxin biosynthesis, sclerotial development, and virulence in both plant and insect hosts, revealing that basic carbon acquisition pathways can be leveraged for multitarget interventions. The environmental roots of resistance are illuminated by Zhang et al., who demonstrate that the agricultural azole uniconazole triggers pan-azole cross-resistance in *C. neoformans* via efflux pump hyper-expression and chromosomal duplication. Their work provides a mechanistic link between agrochemical exposure and clinical therapy failure, reinforcing calls for One-Health stewardship of antifungal compounds. Finally, Suo et al., decode the RNA-binding protein Puf4 as a metabolic gatekeeper *in C. neoformans*; its over-expression rewires carbohydrate storage, elevates ROS, attenuates capsule and melanization, and markedly reduces virulence, offering a rare gain-of-function mutation that can be exploited for live-attenuated vaccine development.

## Reviews: translating insight into intervention pipelines

Complementing these experimental studies, eight reviews synthesize the field’s rapidly moving frontiers. RNA interference, long considered inaccessible in C. albicans, is re-evaluated as a programmable route to silence essential and resistance genes (*ERG11*, *CDR1*, *GSC1*) with chemically modified siRNAs delivered by nanoparticle platforms. Pan-pathogen challenges—toxicity spectra, resistance breakpoints, and drug-drug interactions—are systematically mapped, emphasizing combination and biotech-derived modalities. A comprehensive survey of next-generation chemotypes catalogs agents targeting membrane sterols, cell-wall glucans, DNA replication, and redox homeostasis, while candidly addressing pharmacokinetic gaps and regulatory hurdles. Mitochondrial genomics emerges as a unifying theme linking respiration, azole tolerance, and virulence across *Candida*, *Cryptococcus*, and *Aspergillus*, spotlighting organelle-specific inhibitors as an underexplored strategy. Host-centric perspectives are provided by reviews on cryptococcal immune evasion and the tantalizing, yet still nascent, role of antifungal immunotherapy in reversing T-cell exhaustion and enhancing monoclonal efficacy. Complementing these strands, two forward-looking treatises address: one dissecting how artificial intelligence—deep learning, generative chemistry, and multi-omics integration—can compress the decade-long antifungal discovery pipeline, and another proposing that fungal modulation of the tumor micro-environment warrants adjunctive antifungal strategies in oncological care.

## Toward a cohesive antifungal roadmap

Taken together, the Topic reframes fungal pathogenesis as a systems-level problem in which metabolic wiring, environmental resistance reservoirs, host immunity, and biofilm architecture converge. The original articles furnish mechanistic entry points—natural-product scaffolds, nutrient transporters, RNA-binding proteins, and efflux regulators—while the reviews contextualize these discoveries within therapeutic, technological, and ecological landscapes. We hope this integrative snapshot equips researchers, clinicians, and policy makers with the conceptual and practical tools required to accelerate the next wave of antifungal innovations and, ultimately, to reduce the unacceptably high morbidity and mortality inflicted by these formidable pathogens.
